# Data management plan and REDCap mobile data capture for a multi-country Household Air Pollution Intervention Network (HAPIN) trial

**DOI:** 10.1177/20552076241274217

**Published:** 2024-08-21

**Authors:** Shirin Jabbarzadeh, Lindsay M Jaacks, Amy Lovvorn, Yunyun Chen, Jiantong Wang, Lisa Elon, Azhar Nizam, Vigneswari Aravindalochanan, Jean de Dieu Ntivuguruzwa, Kendra N Willams, Alexander Ramirez, Michael A Johnson, Ajay Pillarisetti, Thangavel Gurusamy, Ghislaine Rosa, Anaité Diaz-Artiga, Juan C Romero, Kalpana Balakrishnan, William Checkley, Jennifer L Peel, Thomas F Clasen, Lance A Waller

**Affiliations:** 1 Department of Biostatistics and Bioinformatics, Rollins School of Public Health, Emory University, Atlanta, GA, USA; 2 Global Academy of Agriculture and Food Systems, University of Edinburgh, Edinburgh, UK; 3 Gangarosa Department of Environmental Health, Rollins School of Public Health, Emory University, Atlanta, GA, USA; 4 Department of Environmental Health Engineering, Sri Ramachandra Institute for Higher Education and Research (Deemed University), Chennai, India; 5 Eagle Research Centre Limited, Kigali, Rwanda; 6 Division of Pulmonary and Critical Care Medicine, Johns Hopkins University School of Medicine, Baltimore, MD, USA; 7 Center for Health Studies, 34850 Universidad del Valle de Guatemala, Guatemala City, Guatemala; 8 Berkeley Air Monitoring Group, Berkeley, CA, USA; 9 Environmental Health Sciences, School of Public Health, University of California, Berkeley, CA, USA; 10 4591 University of Liverpool, Liverpool, England; 11 Center for Global Non-Communicable Disease Research and Training, School of Medicine, Johns Hopkins University, Baltimore, MA, USA; 12 Department of Environmental and Radiological Health Sciences, 3447 Colorado State University, Fort Collins, CO, USA

**Keywords:** Data collection, household air pollution, REDCap, REDCap mobile app, HAPIN, digital data capture, data management, multi-country

## Abstract

**Background:**

Household air pollution (HAP) is a leading environmental risk factor accounting for about 1.6 million premature deaths mainly in low- and middle-income countries (LMICs). However, no multicounty randomized controlled trials have assessed the effect of liquefied petroleum gas (LPG) stove intervention on HAP and maternal and child health outcomes. The Household Air Pollution Intervention Network (HAPIN) was the first to assess this by implementing a common protocol in four LMICs.

**Objective:**

This manuscript describes the implementation of the HAPIN data management protocol via Research Electronic Data Capture (REDCap) used to collect over 50 million data points in more than 4000 variables from 80 case report forms (CRFs).

**Methods:**

We recruited 800 pregnant women in each study country (Guatemala, India, Peru, and Rwanda) who used biomass fuels in their households. Households were randomly assigned to receive LPG stoves and 18 months of free LPG supply (intervention) or to continue using biomass fuels (control). Households were followed for 18 months and assessed for primary health outcomes: low birth weight, severe pneumonia, and stunting. The HAPIN Data Management Core (DMC) implemented identical REDCap projects for each study site using shared variable names and timelines in local languages. Field staff collected data offline using tablets on the REDCap Mobile Application.

**Results:**

Utilizing the REDCap application allowed the HAPIN DMC to collect and store data securely, access data (near real-time), create reports, perform quality control, update questionnaires, and provide timely feedback to local data management teams. Additional REDCap functionalities (e.g. scheduling, data validation, and barcode scanning) supported the study.

**Conclusions:**

While the HAPIN trial experienced some challenges, REDCap effectively met HAPIN study goals, including quality data collection and timely reporting and analysis on this important global health trial, and supported more than 40 peer-reviewed scientific publications to date.

## Introduction

As the volume of data generated by scientific studies expands, so does the complexity of capturing, managing, storing, cleaning, and sharing these data. These challenges, increasingly recognized by funding entities, resulted in the requirement of detailed data-management plans within grant proposals and, most recently, expanded these to provide data management and sharing details.^1^ In the sections below, we detail our experiences implementing a comprehensive data management plan to support the Household Air Pollution Intervention Network (HAPIN) trial,^2^ a large-scale health research study spanning four low- and middle-income countries (LMICs), which investigated the potential health benefits of replacing traditional biomass burning cook stoves with liquefied petroleum gas (LPG) cook stoves.

## The HAPIN trial and its significance

Biomass stoves represent a primary source of cooking energy in many parts of the world, particularly in LMICs, where more modern energy sources are often unavailable or unreliable.^3^ Past research links high levels of household air pollution from biomass stoves to respiratory illnesses and other health problems, particularly impacting women and children who are typically the most exposed to the resulting smoke.^4,5^ According to the World Health Organization's latest report on household air pollution, approximately 3.2 million premature deaths per year are associated with household air pollution caused by the incomplete combustion of solid fuels.^6^

These alarming health implications motivated the HAPIN trial, a large-scale randomized controlled study based in Guatemala, India, Peru, and Rwanda. The detailed design, protocols, and analysis methodologies applied to HAPIN have been published elsewhere.^7–9^ HAPIN aimed to investigate the impact of an LPG stove and fuel intervention on four primary health outcomes, namely, birth weight,^10,11^ growth stunting, severe pneumonia in infants,^12^ and systolic blood pressure in non-pregnant adult women living in the same household as pregnant women.^13^ Other datasets gathered in this trial comprised behavioral survey responses,^14–16^ personal and environmental exposure metrics,^17,18^ stove use temperature traces,^19^ imaging data,^20^ and biosamples.^8^

## The shift towards electronic data capture in health research

Effective data collection in health research is essential, forming the foundation for analyzing and distilling meaningful insights.^21^ Traditionally, health research relied on paper-based methods for data collection, especially in resource-limited settings. However, electronic data collection has increasingly become the norm,^22^ even in resource-limited settings, offering numerous benefits including (but not limited to) improved data quality, increased efficiency, real-time monitoring, and enhanced security.^23,24^ Electronic data capture systems also provide scientists with enhanced data collection and management capability, including tools for scheduling and monitoring data collection, conducting quality control, generating reports, and providing data access to investigators for cleaning and analysis.^23,25^

## REDCap—a solution for efficient research data management

Research Electronic Data Capture (REDCap) is a robust tool developed in 2004 at Vanderbilt University for the secure, web-based capture and management of research data. Its user-friendly interface allows researchers to design custom data entry forms, generate reports and audit trails, and store data in a secure cloud-based repository.

Initially developed to meet the data collection needs of a small group of scientists, REDCap has rapidly grown in popularity. It has been adopted by over 6000 institutions in 153 countries, used in 1.7 million projects, and cited in more than 30,000 peer-reviewed publications.^26^ Importantly, REDCap complies with essential security and privacy regulations, including the US Health Insurance Portability and Accountability Act (HIPAA) and the Federal Information Security Management Act of 2002 (FISMA). In addition, REDCap complies with the privacy and confidentiality requirements of HAPIN's funders (NIH and the Bill and Melinda Gates Foundation).

The HAPIN Data Management Core (DMC) chose REDCap as the primary data collection and management tool. Although there is no single blueprint for a good data management plan, our experience with a large-scale project like HAPIN, with its diverse study sites, local data management teams, and various sets of data, allows us to tell a comprehensive and compelling story about our data management experiences, challenges, and successes that could prove helpful for other research teams and data managers in preparing their own plans.

## Methods

### Study overview

This randomized control trial aimed to reduce household air pollution by providing cleaner fuel for pregnant women in LMICs who use biomass fuels for daily cooking. We enrolled 3200 pregnant women, 800 in each country (Guatemala, India, Rwanda, and Peru), who met the inclusion criteria: 18 to <35 years old, 9 to <20 weeks gestational with a viable singleton pregnancy, used mostly biomass fuel for cooking, and agreed to participate in the study with informed consent. We excluded them if they were smokers (self-reported) or planned to move from the study area in the next 18 months. In about 20% of households overall, other adult women (40 to <80 years of age) who lived in the same household as the pregnant women were recruited as well. After baseline data collection, we randomized their households to intervention and provided an LPG stove and continuous supply of LPG fuel delivered to their home for 18 months, and the control group continued using mainly biomass fuel for cooking.

### Study aims

The HAPIN study aimed to investigate the health effects of using cleaner cooking fuel, LPG, on pregnant women, infants, and other adult women in their households. We hypothesized that the intervention group in all study sites would have offspring with higher birth weights, lower severe pneumonia incidence, and less stunting at 12 months of age compared to the control group. We also hypothesized that the other adult women in the intervention group would have reduced blood pressure after 18 months.

### Statistical analysis

The study design, protocol, and statistical analysis plans have been published in more than 40 HAPIN publications previously and specifically in detail by Clasen, Barr, and Johnson et al.^7–9^

**Figure 1.**
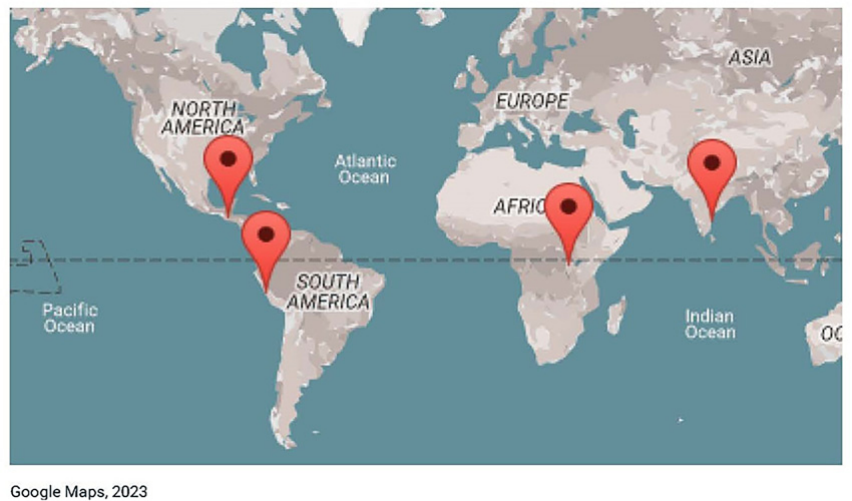
Study site map.

Data collection in remote areas is always challenging, especially in a large multi-country longitudinal study, such as HAPIN (Figure 1). We required a plan suitable for implementation in four countries, including different study sites within some countries. The variety of study sites across HAPIN required the creation of forms in local languages: English, Kinyarwanda, and Guatemalan and Peruvian variants of Spanish. The need for local language compatibility raised additional challenges regarding communication and training impacting data management, as detailed below.

In remote areas with no internet and intermittent electricity, paper data collection is often an acceptable alternative. However, in HAPIN, the volume of data we planned to collect at baseline and multiple follow-up visits (including intervention fidelity and adherence, exposure monitoring, clinical and behavioral outcomes, etc.) and the diverse data types (including surveys, imaging, real-time stove use monitors, exposure monitors, and biospecimens) suggested that paper data collection was not feasible. Given the operational and logistical limitations in remote areas, we needed a secure electronic data capture and management system to safely collect and store the surveys and metadata on other types of collected data.

All study sites were in rural areas consisting of small villages with similar economic status. Our general goal was to keep the data collection as homogeneous as possible across the sites. However, due to the nature of the study (and its emphasis on cooking, a highly culturalized activity), we had to accommodate the cultural and social differences in our study population, particularly regarding cooking behavior. This required that some of our nutritional and behavioral CRFs had slightly different questions in different countries.

Moreover, the HAPIN study investigators were from multiple academic institutions in the US, the UK, and each study country. This scenario required secure (blinded where necessary) and ongoing read-only access to the data for logistical planning, study progress monitoring, and data completeness and quality reporting. This approach was only possible on a digital platform with multi-user access capability.

## Mobile data collection device selection

The HAPIN study required a mobile data collection device that would function with the REDCap mobile application in the study sites, meeting the criteria below:

- *Long battery life*. This was one of the project's most important selection factors. The data collection teams would leave the local study center early in the morning and work in the field until the end of the day, with little or no time or opportunity to charge the devices. We needed to select a device that could function for many hours without recharge until the team could return to their centers.
- *Affordable cost*. Considering the dimensions of the HAPIN study, we required tens of tablets at each site and extra devices in case of device failure.
- *Reasonable size/lightweight*. Our study settings involved interviews in study participants’ rural homes. As a result, most data collection occurred while the study staff were standing with a tablet in their hands. We desired a lightweight device, considering possible staff fatigue after long hours of data collection.
- *Camera*. We planned to use barcodes for study/participant IDs and for identifying collected biosamples and personal air pollution exposure devices used during the exposure assessment visits. The device camera and the barcode scan function were essential.
- *Availability*. Considering the duration of the study (5 years), we had to select a device that would be on the market for at least a few more years and also be available in each of our study countries’ markets.

Based on these criteria, we selected the Samsung Galaxy E tablets manufactured by a global brand (Samsung Electronics Co., South Korea), which had stable/sustainable support in each of our study countries.

### Case report form (CRF) development and documentation

**Figure 2.**
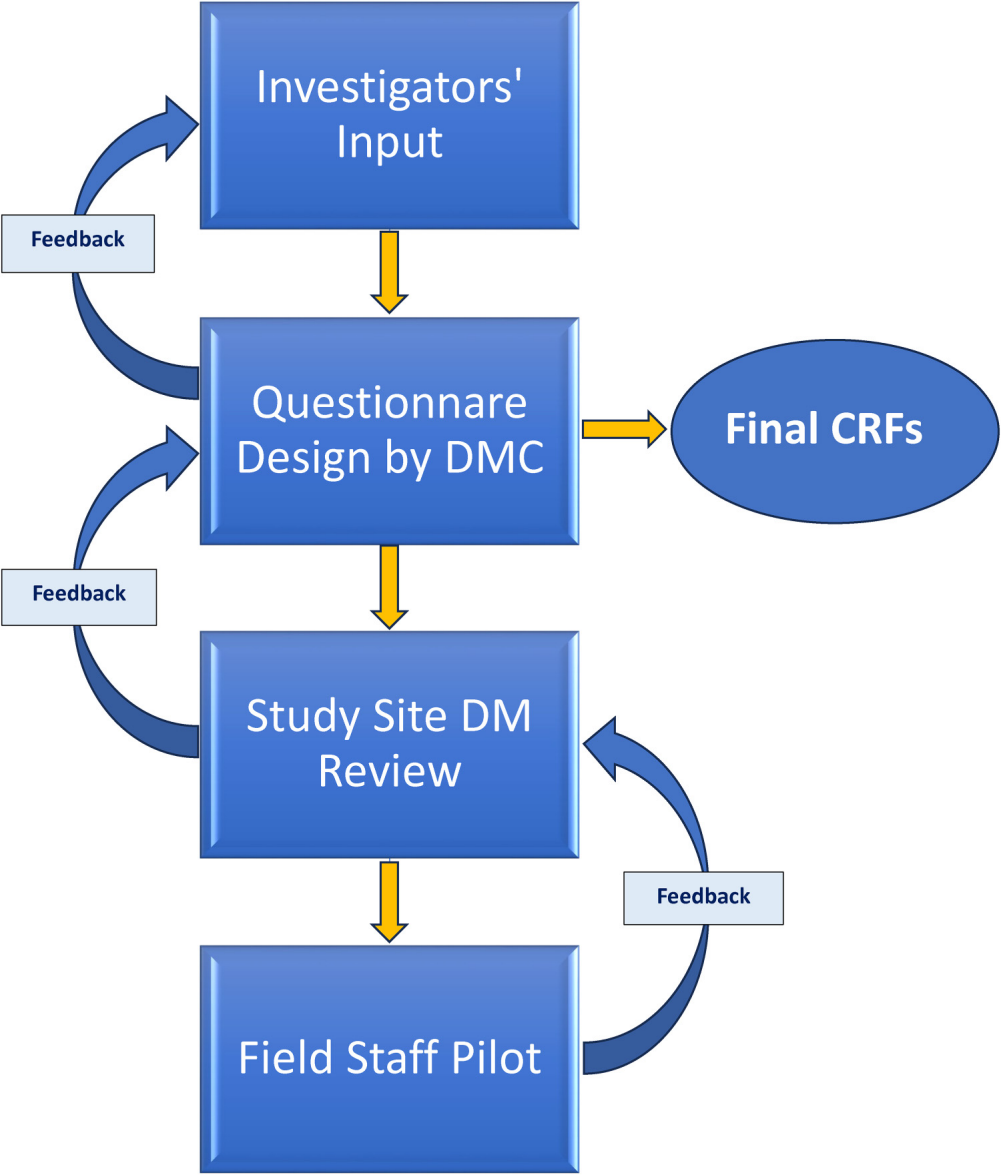
Case report form (CRF) design flow.

In collaboration with HAPIN investigators, DMC, and local study teams, we developed over 80 unique CRFs to capture the following information: (1) household: household structure and family cooking behavior; (2) pregnant woman: pregnancy status and outcome (up to and including birth); (3) mother (previous pregnant woman): health, nutritional status, and activities; (4) children: health and development (following birth); (5) non-pregnant adult woman in the household (if any): health and activities; and (6) household: information on installation and uninstallation of air pollution exposure and stove use monitoring devices,^7^ and LPG stove and tank deliveries/repairs. The CRF development process involved frequent (generally weekly) interaction between the DMC and HAPIN investigators as well as with local study teams, which occurred mostly virtually (Figure 2). The DMC's goal was to harmonize CRFs as much as possible without ignoring study communities’ differences and sensitivities.

Although all CRFs were drafted in English, the local study teams translated and utilized them in their local languages. The translated CRFs were back-translated to English to assess translation quality and compared to the original drafts. This lengthy but critical process helped teams identify and correct any mistranslations.

In India, the team communicated with the participants in the local language but used the English CRFs for data entry in REDCap. In Rwanda, most CRFs were translated to Kinyarwanda and used for oral communication and data entry. As noted above, the Guatemala and Peru sites used the Spanish translation of CRFs, with some local dialect customizations, particularly regarding terms for cooking.

### ID system and CRF coding

According to the HAPIN research protocol,^9^ each household could include one to three participants throughout the follow-up period. Instead of using a standard participant-based ID to collect and track data, we implemented household identifiers (HHIDs). We collected information on each household and its participants using one identifier. The HHID worked as an umbrella that linked all observations from one household, including the personal data on the pregnant woman (later mothers), non-pregnant adult woman, and child, as well as the household physical and stove use characteristics. We also made a laminated ID card with a QR code for each HH, which the staff simply scanned with the tablets to start data collection for that specific HH. This reduced possible HHID entry errors during data collection. To differentiate the types of information collected within each household, we used different “series” of CRFs delineated by a lead alphabetical character representing which individual within a household was the focus of a particular CRF. As a result, the HAPIN M, A, C, and H CRF series were associated with mother, non-pregnant adult woman, child, and household, respectively. Each CRF coded by the series letter and two-digit number. For example, M13 was created to collect pregnant women's health status and C33 for child health status. This coding proved invaluable for facilitating communications throughout the study, especially across study sites. For example, the “M11-Lifestyle Behaviors Questionnaire” is “M11-Comportamiento Sobre Estilo De Vida” in Spanish and “M11-Ibibazo ku myitwarire y'uburyo bwo kubaho” in Kinyarwanda, but we simply used the CRF code to address it. When implementing the CRFs in REDCap, we also used these codes on variable nomenclature, detailed below.

### Training

One of the challenges of electronic data capture is staff training. In addition to training on the standardized administration of each CRF, HAPIN study staff required training on REDCap, the REDCap mobile app, and tablet use. The central DMC staff provided in-person training via hands-on workshops targeting each country's local data management team and field staff. At the end of the workshops, the team dedicated a day to a dry run of data collection, mainly in the field, to make any last adjustments.

The DMC also created training videos on tablet use and the steps to connect to the REDCap website. Each video explained and demonstrated step-by-step instructions for data collection on tablets, data upload from the tablets, checking the status of uploaded data on the REDCap webpage, etc. The training materials and documents were all in English, but each training session was translated into local languages. We also created and distributed standard operating procedures for data collection, data upload, and tablet maintenance to ensure all sites implemented standardized processes.

## Implementation and data collection

### Implementation

**Figure 3.**
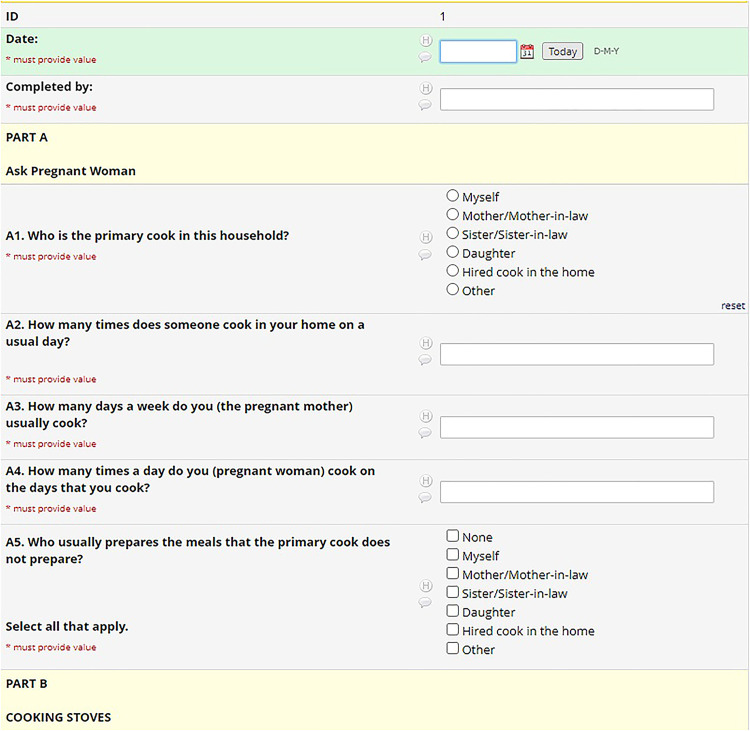
English CRF.

**Figure 4.**
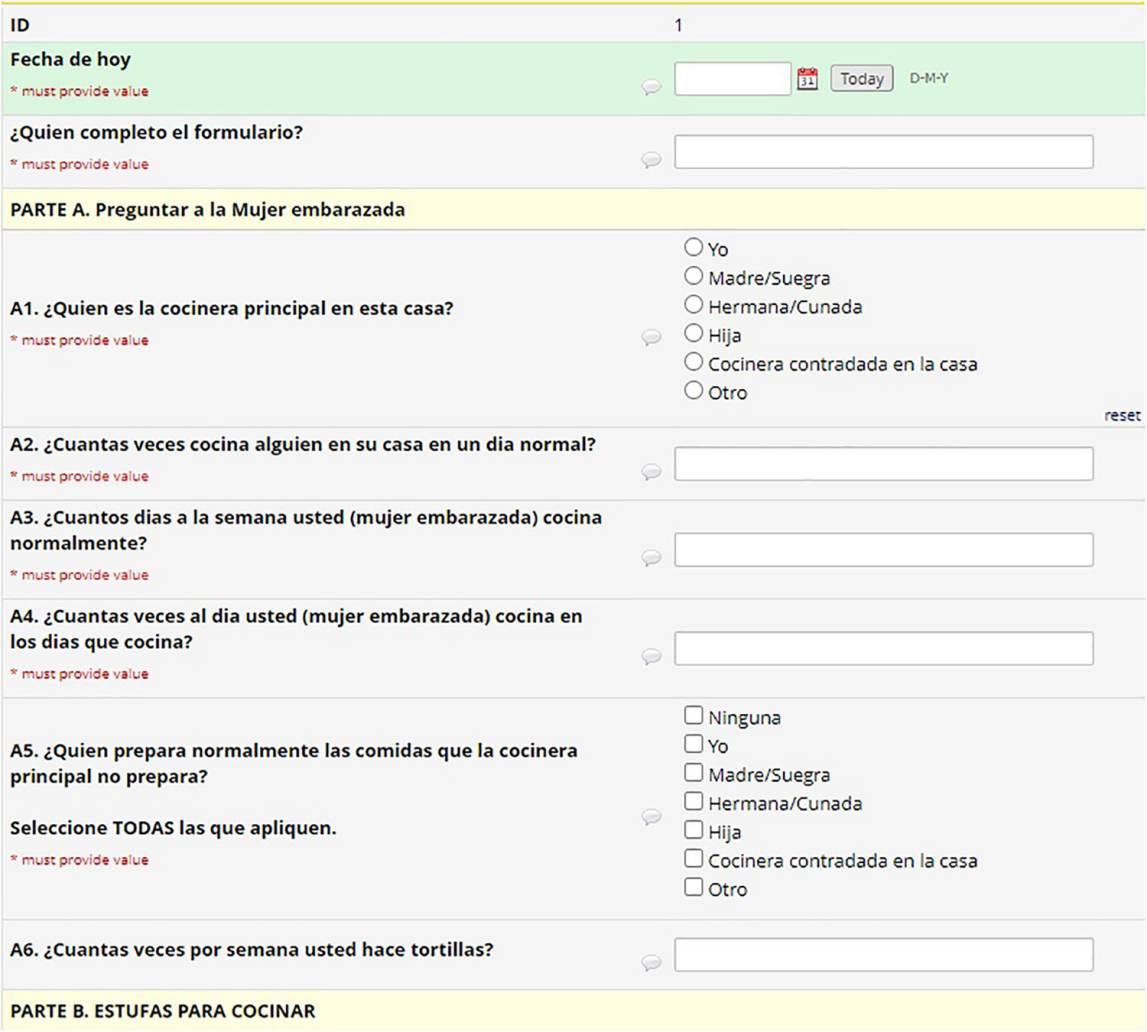
Spanish CRF.

**Figure 5.**
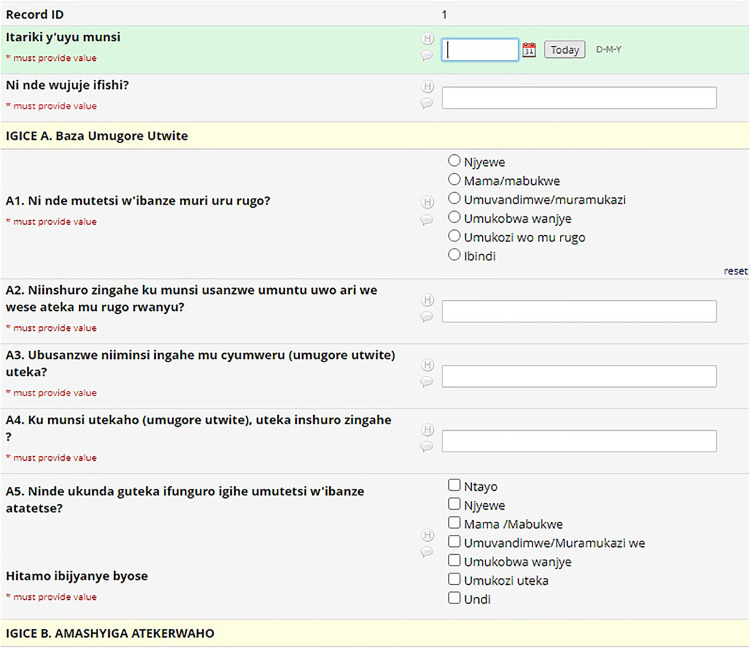
Kinyarwanda CRF.

As explained previously, the coding system (M, A, C, and H) defined variable nomenclature. The REDCap system uses a flat-file database that requires unique names for all 4000 variables in the HAPIN study. To maintain each variable's uniqueness and to link variables to their parent CRFs, a variable name on each CRF started with that CRF's code (e.g. M11_date). We created a separate project for each country in REDCap and kept the variable names the same, but translated the labels (questions) into each country's local language (Figures 3 to 5). This naming protocol allowed local teams to view the questions in their local language. Still, it kept the backend of all systems the same, facilitating all four countries’ data integration for reporting and analysis purposes. Recently, REDCap released the multi-language module, which allows users to add multiple languages and select one from the drop-down menu. This would be helpful for smaller projects, but on a project with HAPIN's dimensions, it was better to collect and keep the data for each country in separate projects. Later in this article, we explain the maintenance and data export issues for large projects, which we assume will be magnified on more extensive projects.

Since variable names were the same across study locations, we required a system to identify the region of the collected data after integration. The HHID ranges worked well for this purpose. We assigned separate ranges of HHIDs to each country to avoid misidentification by slight typographic errors. Our five-digit IDs started with 1 in India (e.g. 13000), 2 in Rwanda, 3 in Guatemala, and 4 in Peru.

The REDCap application includes data quality checks and verification functionality to ensure the data entered are accurate and complete. We used these validation rules and functions to automate data quality control where possible. If the field staff entered an out-of-range date or an answer in the wrong format, the REDCap mobile app warned them via popup messages that supported real-time correction. In addition, designating a variable as “Required” prompted the system to demand data entry when the field was blank before saving and moving to another form. The branching logic function also helped save field staff time and effort when it was applicable.

Furthermore, we used identical codes for the common answer options in multiple-choice questions like “Other, specify,” “Don't know,” or “Not applicable.” For example, using “555” as the code for all “Other, specify” answer options facilitated the data quality processes when we needed to check all the collected text data for multiple-choice questions. Once we implemented these CRFs in REDCap, they could be downloaded and shared, providing a strong base for future related research.

### Data sources and integration

The HAPIN field staff visited each household 30 times on average. Again, to manage and distinguish these visits, we used a coding system, including a letter and consecutive numbers for visits. For example, pregnancy visits were called P1 and P2, and we used B1, B2, B3, and B4 for post-birth visits. As a part of data collection, over 55,000 biosamples (e.g. dried blood spots and urine samples) were collected during different visits from all study participants (pregnant women, non-pregnant adult women, and children) to measure various exposure-related biomarkers.^8^ We used a nomenclature format that included the HHID, sample type (B, U), participant type (M, O, C), and visit (P1, P2, etc.) segments to create a unique identifier for each sample. Then, we made pre-printed barcode labels based on the identifiers to mark the biosample containers. The staff scanned the barcode during data collection and marked the collection time and other related information in the designated CRF. We used similar naming conventions and processes for instrumental exposure measurements, and these facilitated the data integration of biomarker measurement results to the sample collection metadata and other exposure measures.

**Figure 6.**
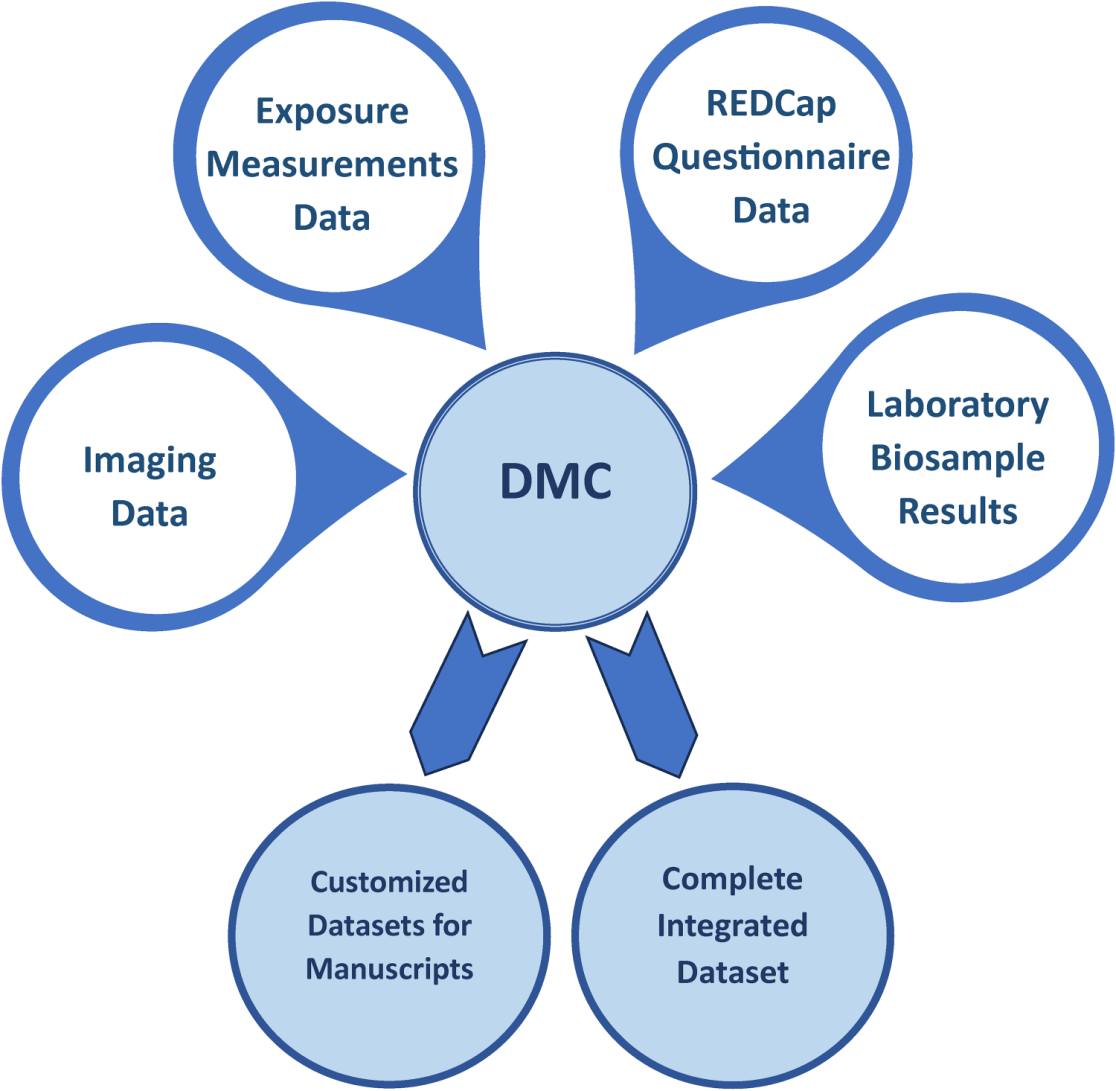
Data integration flow.

Furthermore, ultrasound images were collected during the study from pregnant women, non-pregnant adult women, and children to follow the fetus' growth, measure blood vessel thickness and elasticity, and confirm a pneumonia diagnosis, respectively.^27^ We used a similar naming format to identify and integrate imaging data with metadata collected in REDCap (Figure 6).

### Scheduling

**Figure 7.**
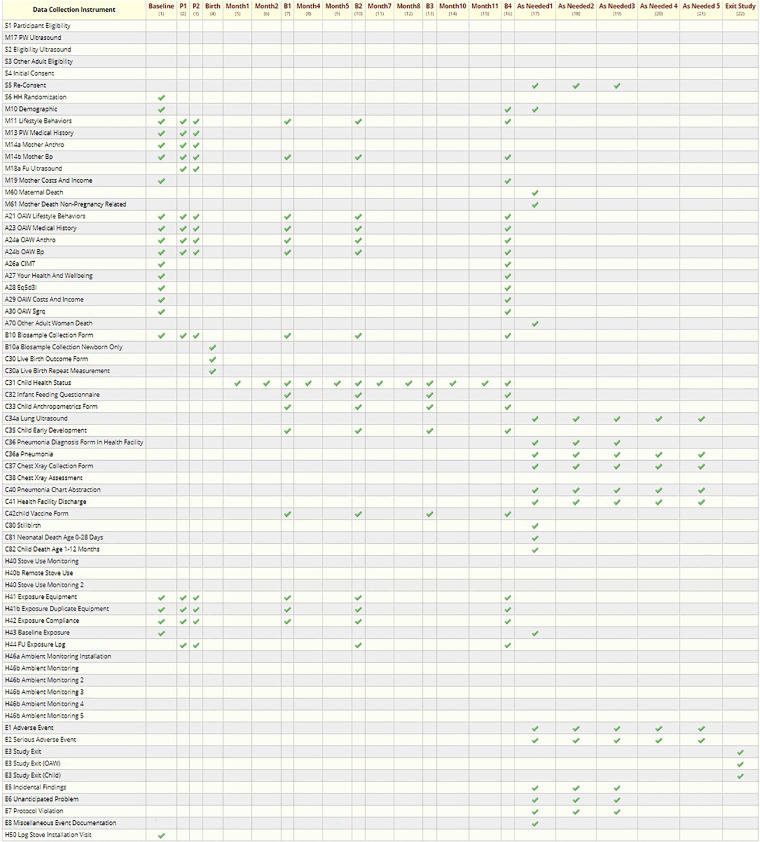
Main visits data collection schedule (REDCap screenshot).

**Figure 8.**
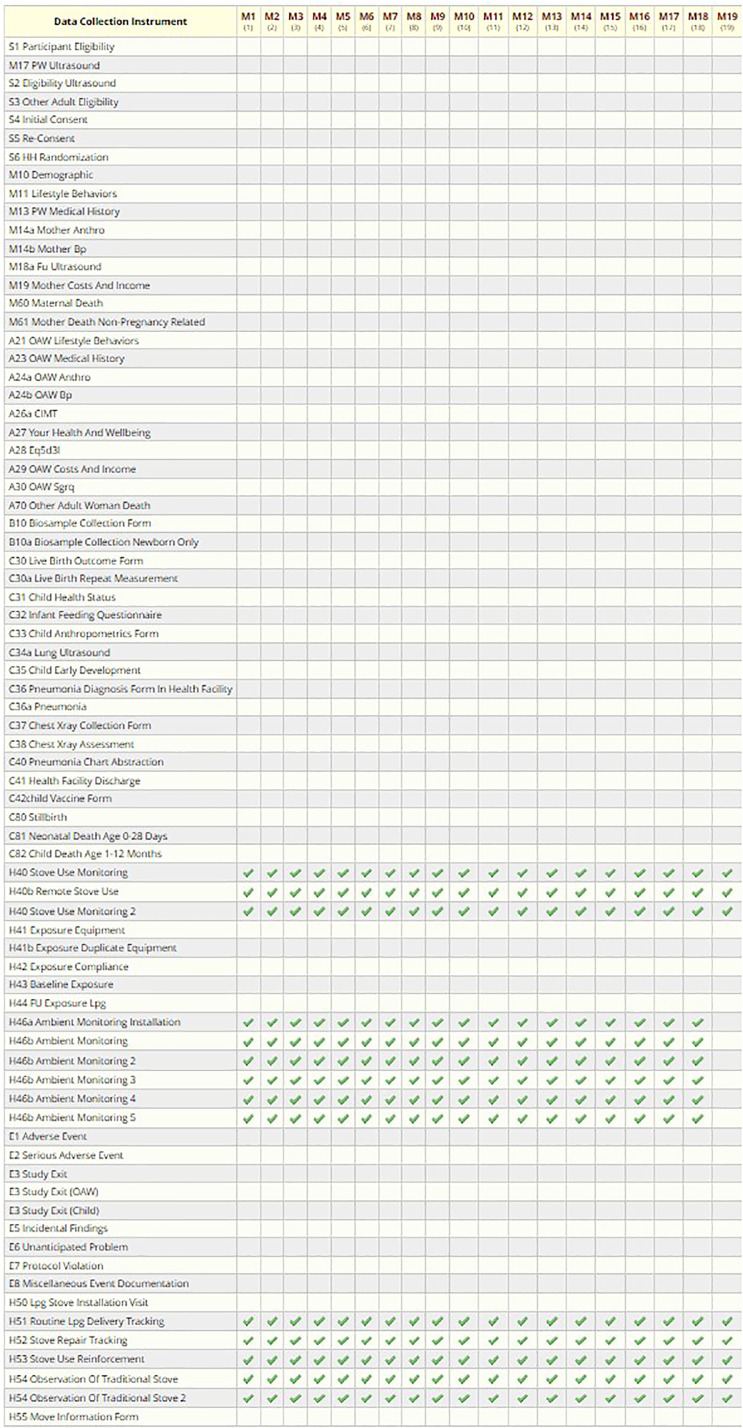
Monthly visit data collection schedule.

The HAPIN DMC defined the study events (visits) and study arms (based on visit timeline, not based on intervention) in REDCap and assigned the appropriate CRFs to each visit to facilitate precise data collection based on the study protocol. After screening and recruitment, we collected data in two different timelines. The first one, referred to as the “Main Study,” involved nine scheduled visits, generally 3 months apart (birth-related) (Figure 7 and Table 1). The second timeline, “Monthly Visits, “ included about 19 visits, each 1 month apart, and was not associated with the birth date (Figure 8 and Table 2).

**Table 1.** Schedule of the main study visits’ data collection of exposure and the health outcome.

Data collection instruments (CRFs)	**Eligibility**	**Baseline**	**P1 (24–28 W)**	**P2 (32–36 W)**	**Birth**	**B1 (3 months after birth)**	**B2 (6 months after Birth)**	**B3 (9 months after Birth)**	**B4 (12 months after Birth)**	**As needed**
S1—Initial Eligibility	X									
M17—Ultrasound	X									
S2—Ultrasound Eligibility	X									
S3—OAW Eligibility	X									
S4—Consent	X									
S6—Randomization		X								
M10—Demographic		X							X	
M11—Mother Lifestyle		X	X	X		X	X		X	
M13—Mother Medical		X	X	X						
M14a—Mother Anthro		X	X	X						
M14b—Mother BP		X	X	X		X	X		X	
M19—Mother Cost/Income		X							X	
M60—Maternal Death										X
A21—OAW Lifestyle		X	X	X		X	X		X	
A23—OAW Medical		X	X	X		X	X		X	
A24a—OAW Anthro		X	X	X		X	X		X	
A24b—OAW BP		X	X	X		X	X		X	
A26a—OAW CMIT		X							X	
A27—OAW Health		X							X	
A28—OAW EQ-5D		X							X	
A29—OAW Cost/Income		X							X	
A30—OAW SGRQ		X							X	
A70—OAW Death										X
B10—Biosample		X	X	X	X	X	X		X	
C30—Live Birth					X					
C31—Child Health						X	X	X	X	
C32—Infant Feeding						X	X	X	X	
C33—Child Anthro						X	X	X	X	
C35—Child Development						X	X	X	X	
C42—Child Vaccination						X	X	X	X	
C80—Child Death										X
H41—Exposure Equipment		X	X	X		X	X		X	
H42—Expo. Compliance		X	X	X		X	X		X	
H43—Baseline Exposure		X								
H44—FU Exposure			X	X		X	X		X	
H50—LPG Installation		X								
E2—Adverse Event										X

**Table 2.** Monthly visits’ data collection schedule.

Instruments	M1	M2	M3	M4	M5	M6	M7	M8	M9	M10	M11	M12	M13	M14	M15	M16	M17	M18	M19
H40—Stove Use Monitoring	X	X	X	X	X	X	X	X	X	X	X	X	X	X	X	X	X	X	X
H40b—Remote Stove Use Monitoring	X	X	X	X	X	X	X	X	X	X	X	X	X	X	X	X	X	X	X
H46—Ambient Monitoring (weekly, ×5)	X	X	X	X	X	X	X	X	X	X	X	X	X	X	X	X	X	X	X
H51—LPG Delivery Tracking	X	X	X	X	X	X	X	X	X	X	X	X	X	X	X	X	X	X	X
H52—Stove Repair Tracking	X	X	X	X	X	X	X	X	X	X	X	X	X	X	X	X	X	X	X
H53—Stove Use Reinforcement	X	X	X	X	X	X	X	X	X	X	X	X	X	X	X	X	X	X	X
H54—Observation of Traditional Stove Use	X	X	X	X	X	X	X	X	X	X	X	X	X	X	X	X	X	X	X

**Figure 9.**
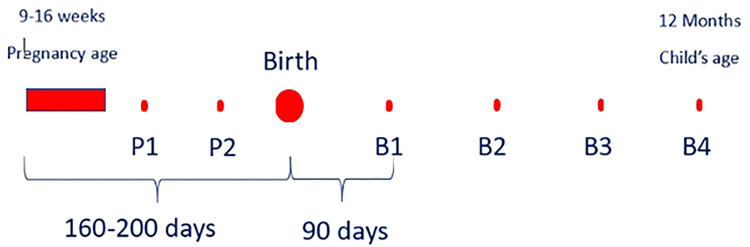
Birth-related visits scheduling module.

Considering the complexity of the HAPIN study design/protocol across the study sites, defining an overall scheduling module proved particularly challenging. In many studies, the day of the subject recruitment is labeled “day 0” (or “day 1”), and the final study visit is “day n”. In the HAPIN study, pregnant women were recruited within an eligibility window of 9–20 gestational weeks, so the baby's birth could happen at any time within a roughly 30-week window after recruitment. There were two scheduled visits after the baseline visit based on the pregnant woman's gestational week. The follow-up period after the child's birth consisted of the next 12 months. Therefore, since birth reflected a significant time point of the study, the DMC marked the estimated birth date, rather than the recruitment date, as “day 0” in the scheduling module. Thus, the recruitment day could be a range of days (−200 to −140 days) before birth. Once the actual date of birth was recorded in the REDCap scheduling system, all follow-up visits after the birth were adjusted, marking the birth date as day 0 and following visits as +30 days,  + 90 days, etc. (Figure 9).

**Figure 10.**
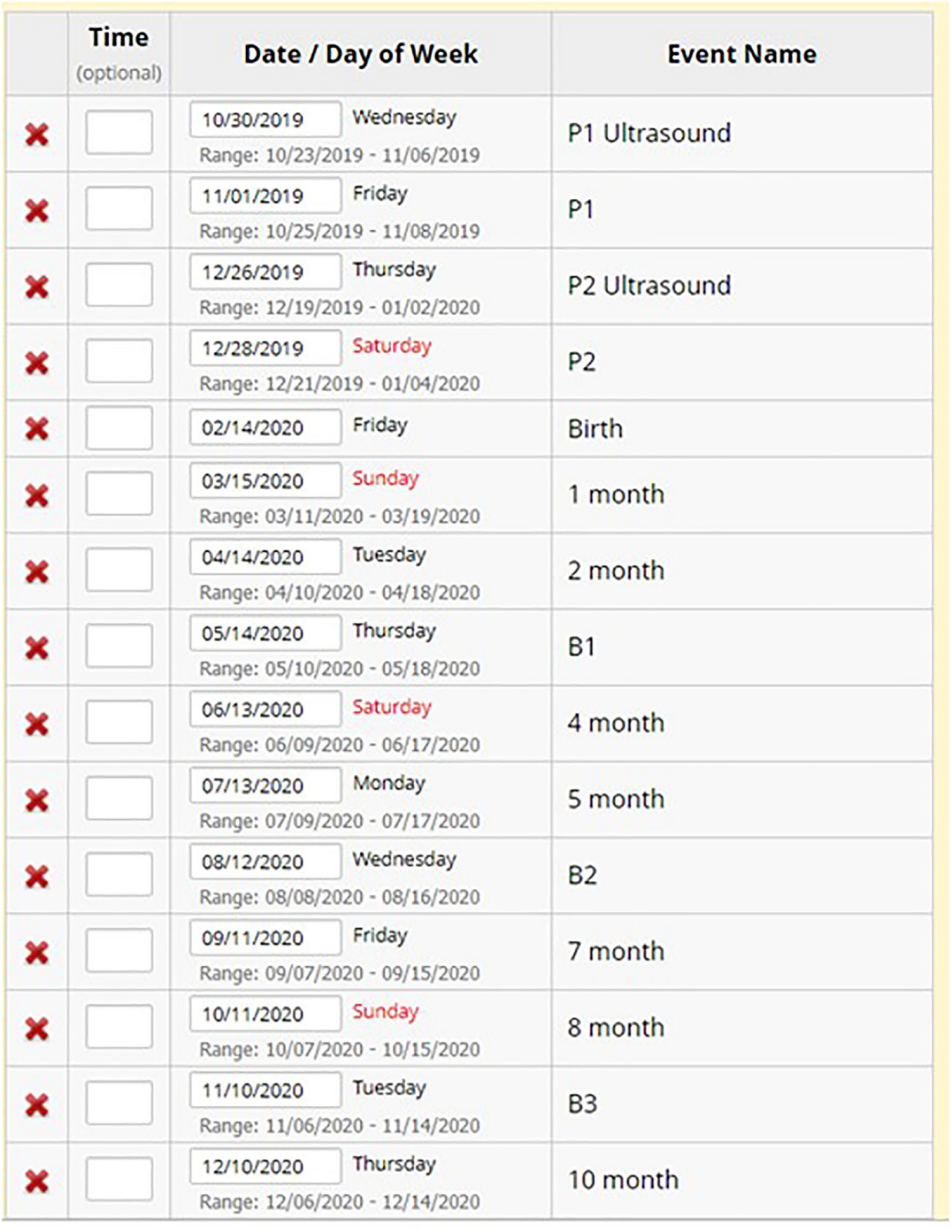
Household scheduled visits in REDCap.

The second HAPIN study visit timeline involved monthly household visits, specifically monitoring cooking behaviors, LPG delivery, and stove repairs in intervention households. We expected each intervention household to have 16–19 monthly visits after the baseline visit. These visits were unrelated to the gestational age or birth date, so they were scheduled at the beginning of the study based on the recruitment day or when the household needed an LPG delivery (Figure 10).

**Figure 11.**
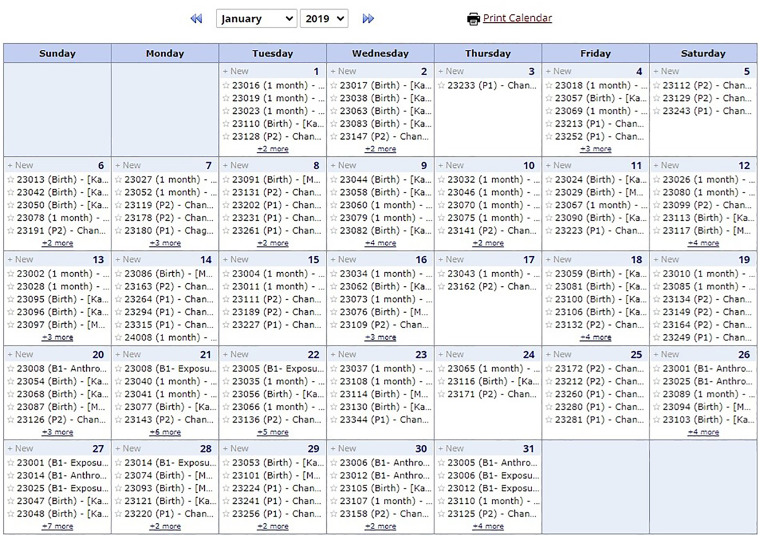
Study site scheduled visit calendar.

The REDCap calendar function is pivotal in facilitating the smooth execution of longitudinal studies involving mother and child cohorts by assisting in project logistics planning. The HAPIN scheduled visits and data collection points ensured precise and timely data acquisition and provided a visually intuitive format. This feature helped optimize resource allocation, personnel coordination, and overall project management and enhanced communication and collaboration among the research teams, enabling them to proactively anticipate and address logistical challenges (Figure 11).

### Randomization

The randomization procedures developed by the DMC were designed to be consistent and independently applied at each study site. The DMC generated randomization lists based on block randomization using randomly selected block sizes.^28^ This approach can achieve balance when assigning participants to treatment groups and avoid selection bias. We generated different lists for different study sites within each study country. To earn participants’ trust in the randomized treatment assignment process, we prepared sealed randomization envelopes, each containing a single treatment assignment card, and used the following procedure to assign households to either of the treatment groups: receive an LPG cook stove and continuous fuel provision throughout the study (intervention group) or continue using biomass stove (control group). In each household, the field team laid the first six sealed envelopes (from the ordered stack of envelopes assigned based on the randomization list) on a table and asked the representative from the participating household to select an envelope and open it. Then, that household was assigned to the treatment arm shown on the card in the chosen envelope. This process was monitored and checked using the envelop numbers and the real-time data entered to RC by the DMC to ensure the integrity of the processes at all study sites. All non-opened envelopes returned to DMC and voided after randomization completed.

### Update, maintenance, and problem-solving during data collection

When data collection began, some adjustments to CRFs were necessary based on feedback from local data teams, field staff, and investigators. The REDCap structure allowed the DMC to make the changes (adding, deleting, or revising questions and response options or adding CRFs) with minimal difficulty in a secure way and without adversely affecting previously collected data. REDCap saves the project's revision history by timestamp and a changed data dictionary. This flexibility in REDcap was particularly beneficial when the COVID-19 global pandemic limited in-person data collection. We made necessary changes to CRFs to allow data collection to proceed and remain as compliant as possible with the original protocol. Due to the sites’ geographical location and time zone differences, we defined a consistent, but short weekly window (typically Sunday mornings in the Eastern Time Zone) for system updates to avoid data upload process interruption. Throughout the study, the DMC met (virtually) weekly with study site data management teams to address any concerns, answer questions, announce the latest changes, provide results of data quality reviews, and discuss future changes or adjustments.

It is important to briefly mention some challenges we encountered due to the project's size and nature (e.g. multi-language). Periodically, we had issues with data upload from the tablets, so we had to use the data dump feature and later map the data through the server. Special characters can pose challenges in REDCap, especially when using the mobile application. They may not display correctly or disappear from the text entirely, requiring extra attention and proper handling. This also can cause issues with the data analysis software such as SAS.

Furthermore, data export can also be challenging in a project as extensive as HAPIN. For large datasets, the export can be time-consuming and may result in timeouts, causing incomplete or failed exports. We overcame this problem by exporting data in smaller subsets rather than the entire dataset to reduce the load and time required for the export.

### Quality assurance and quality control

**Figure 12.**
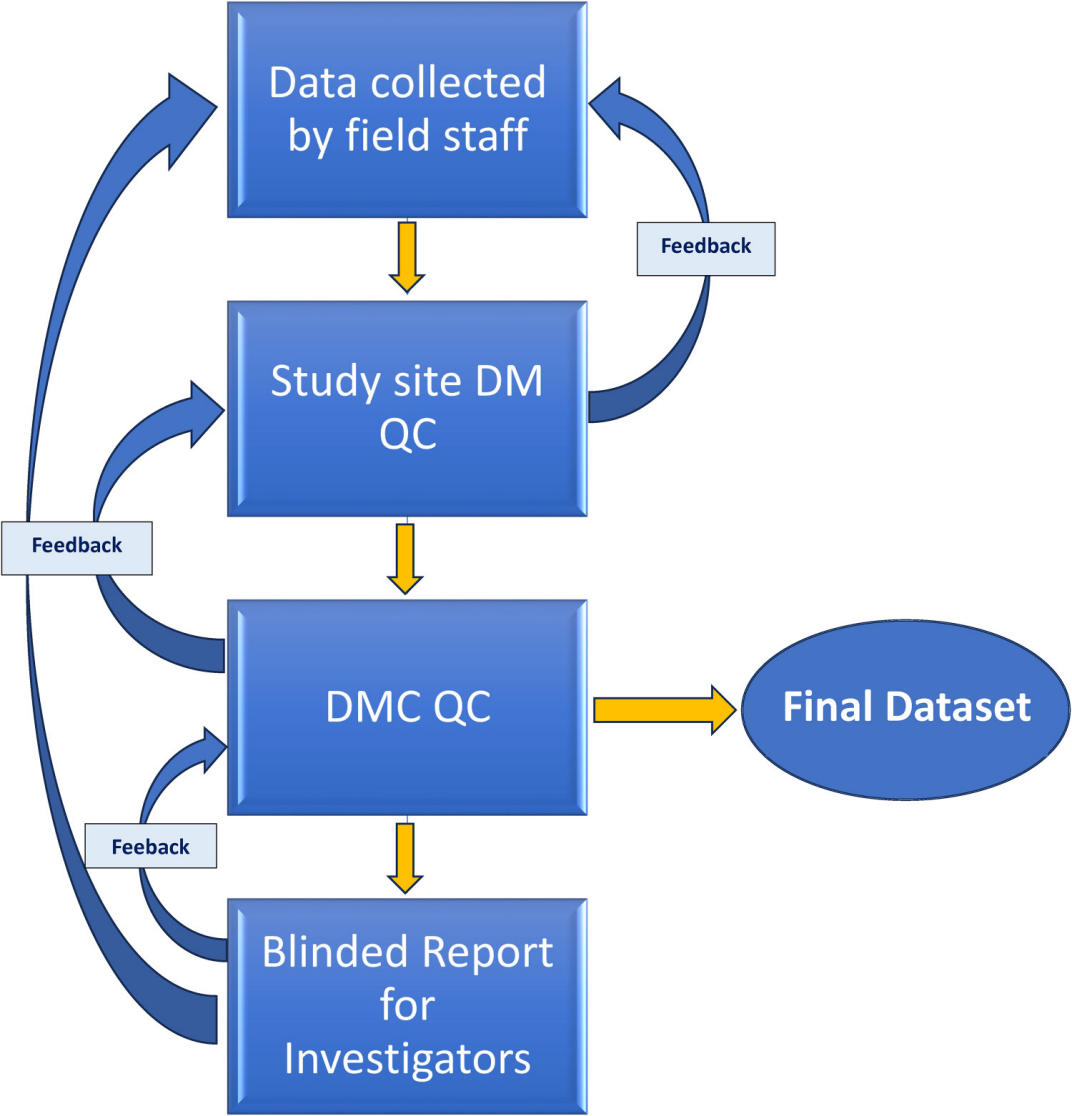
Data quality assurance (QA) and quality control (QC) processes flow.

In addition to the REDCap real-time quality check and the study sites’ data management teams check before and after data uploads, the DMC created monthly data query reports, summarized in an Excel file, for each study site and shared these with local data management teams (Figure 12). These query reports outlined multiple dimensions of HAPIN data quality, including:

- *Completeness.* The DMC created reports to check the completeness of essential questions on each CRF and reported missing data to the local study teams.
- *Timeline (visit window and order)*. The DMC generated reports on data collected outside of the timeline allowed by the study protocol, and by examining the visit dates and validating their order, the DMC identified out-of-order visit dates and reported them to the study sites.
- *Between-CRF checking.* Some questions triggered responses in a separate, detailed follow-up CRF. The DMC tracked the completeness of these relationships, identified any missing CRFs, and listed them in the monthly query reports.
- *Outliers.* DMC examined collected numeric values and ensured they were in a reasonable range, if applicable (e.g. variables, such as blood pressure and anthropometric data). Any suspected outliers were listed in the query report as well. DMC utilized the package “World Health Organization Child Growth Standards SAS igrowup” to calculate age-adjusted z-scores for children's weight, height, and head circumference, examined growth trends, and flagged those which did not follow the anticipated trends.
- *Study site comparisons.* Comparing the four countries’ data helped DMC identify systematic errors in sites needing retraining or extra attention.

### Data sharing

In addition to data collection, management, and quality checks, REDCap also proved valuable in meeting data-sharing needs for HAPIN. The duration and global distribution of the study required real-time access by HAPIN DMC for multiple ongoing analyses, report generation on study progress, adverse/severe adverse events monitoring, and ongoing monitoring of study progress for reporting to the funding agencies. The integrated data generated by local data uploads proved very helpful in providing timely feedback to each constituency. The DMC quickly and reliably linked and combined data and created derived variables consistently applied across study sites. The onset of the COVID-19 pandemic required rapid review, assessment, and planning to maintain data collection and adhere as closely as possible to the original HAPIN protocol. REDCap allowed assessments of progress, projections of ongoing recruitment, and calculations of potential impacts on study power resulting from the pandemic response (particularly limitations in contact between study teams and households).

In order to meet data sharing goals and requirements associated with best practices and required by project funders, the DMC created integrated analytic datasets for each HAPIN publication and presentation approved by HAPIN leadership. Upon presentation or publication, the deidentified and unblinded data are archived in online data repositories (e.g. Dataverse) with persistent digital object identifiers (DOIs), allowing citation and linkage to their accompanying publication. Preserving the research data in a sharable format reduces the risk of duplicate research efforts. Data involving individual identifiers require more care in archiving and providing access, and REDCap features aided the DMC in creating and sharing the deidentified datasets associated with HAPIN research findings.

## Conclusion

Our experience showed that having a central data management team ensures that data collection, storage, and analysis processes are standardized across all study sites. This consistency helps minimize errors and biases, improving the overall quality and reliability of the study results. With a centralized team responsible for data management, there is a streamlined data handling, cleaning, and validation process. This efficiency can accelerate data analysis and reporting, ultimately reducing the overall study timeline. A dedicated data management team often comprises experts in data handling and analysis who can provide training and support to local researchers and study sites, enhancing data collection protocols and data quality along with local capacity building for implementing rigorous data collection and usage. The central team can monitor data collection and quality in near real time, allowing them to identify issues promptly and implement corrective actions, which helps maintain study integrity.

The development and documentation of over 80 CRFs in multiple languages facilitated standardized data collection across diverse study sites and cultural contexts while keeping the variable names consistent across different countries and helped with data comparison and integration. The use of HHIDs and coding systems improved data organization and communication between different study teams and sites.

However, operating a central data management team across multiple countries can present communication and coordination challenges. Time zone differences, language barriers, and cultural variations may impede seamless collaboration. Despite standardization efforts, cultural differences or local practices might introduce biases in data collection or interpretation, especially if not adequately addressed by the central team.

Utilizing the REDCap data collection system proved valuable for managing data in the HAPIN trial. It resulted in the collection of more than 50 million data points and more than 40 published manuscripts in peer-reviewed journals to date.^29–32^ The challenges of collecting data in remote areas were effectively addressed using the REDCap platform, allowing for secure and efficient electronic data capture. The REDCap mobile application allowed for real-time offline data collection and evaluation, which helped provide more accurate and complete data. The platform also included automatic audit trails, data quality checks, and verification procedures to ensure the accuracy of the data collected. REDCap reduced the time and effort required to complete the study by reducing the design and system maintenance and eliminating the need for manual data entry and transfer. The REDCap mobile application used secure login protocols and encrypted data storage to protect the confidentiality of the study participants and the data collected. The platform also included data backup and recovery procedures to ensure that data were lost in the event of a system failure. While the REDCap mobile application proved valuable for HAPIN data collection and management, it involves additional costs, such as purchasing mobile devices, technical support, and training. These costs may impact the overall study budget.

In conclusion, a central data management team in a multi-country research study offers many benefits, such as consistency, data security, and efficiency. However, it also requires careful planning, robust communication, and a clear understanding of local regulations and cultural considerations to overcome the potential challenges.
